# Advances in the study of ARR3 in myopia

**DOI:** 10.3389/fcell.2025.1551135

**Published:** 2025-03-11

**Authors:** Yi-Ming Guo, Junhan Wei, Jiaqi Wang, Guoyun Zhang, Jiejing Bi, Lu Ye

**Affiliations:** Shaanxi Eye Hospital, Xi’an People’s Hospital (Xi’an Fourth Hospital), Affiliated People’s Hospital of Northwest University, Xi’an, China

**Keywords:** ARR3, early-onset high myopia, cone photoreceptor, x-chromosome inactivation (XCI), photosensitive retinal ganglion cells (pRGCs)

## Abstract

The ARR3 gene (cone arrestin, OMIM: 301770) has gained significant attention as a pivotal factor in the etiology of myopia, particularly early-onset high myopia (eoHM). As a member of the arrestin gene family, ARR3 is predominantly expressed in cone photoreceptors, playing a crucial role in visual processing. Recent studies have identified specific mutations in ARR3 that correlate with an elevated risk of myopia development, highlighting its potential involvement in the disease’s pathogenesis. This review summarizes current advancements in elucidating the relationship between ARR3 and myopia, emphasizing genetic variations associated with refractive errors and their implications for myopia research and clinical management. We emphasize the necessity for further studies to elucidate the role of ARR3 in myopia, particularly regarding its impact on visual development and the genetic predisposition observed in specific populations.

## Introduction

Pathological changes in the eyes caused by high myopia (HM) have emerged as one of the major etiological factors for global low vision and vision loss (Verhoeven et al., 2015). With the increasing prevalence of electronic devices and urbanization, the number of people affected by high myopia is projected to rise from 277 million in 2010 to 516 million by 2030, potentially reaching 980 million by 2050 (Baird et al., 2020; Holden et al., 2016). The pathological progression of high myopia involves severe structural changes in the posterior segment of the eye (Jonas and Xu, 2014), such as posterior scleral staphyloma (Ohno-Matsui and Jonas, 2019), myopic choroidal neovascularization (Cheung et al., 2017), maculopathy (Ruiz-Medrano et al., 2019), and retinal detachment (Pierro et al., 1992), frequently leading to irreversible vision impairment. Currently, the pathogenesis of pathological myopia remains poorly understood, and effective interventions are still lacking (Haarman et al., 2020).

Recent genetic studies have revealed key pathogenic mechanisms of myopia, particularly in the context of pathological high myopia. The rapid advancement of sequencing technologies has provided efficient tools for extensive and in-depth research into genetic mechanisms. For instance, linkage analysis and genome-wide association studies (GWAS) have identified nearly 200 gene loci associated with refractive errors and myopia (Tedja et al., 2019). Whole exome sequencing (WES) has facilitated the identification of several genes associated with myopia, including genes related to autosomal dominant inheritance (e.g., ZNF644, SCO2, SLC39A5, P4HA2, BSG, and CDC111), autosomal recessive inheritance (e.g., LEPREL1, LRPAP1, and CTSH), and X-linked inheritance (e.g., OPN1LW and ARR3) (Cai et al., 2019). These findings not only enhance our understanding of the genetic mechanisms underlying myopia but also provide important references for future research.

Mutations in the ARR3 gene, associated with X-linked female-limited inheritance (known as MYP26), are particularly noteworthy. The ARR3 gene is located at Xq13.1, comprises 17 exons, and is involved in the phototransduction and deactivation of cone cells (Murakami et al., 1993). In 2016, Xiao et al. first reported that heterozygous mutations in the ARR3 gene are pathogenic factors in three families with eoHM (Xiao et al., 2016). ARR3 gene mutations had not been linked to any human diseases before, making ARR3 the second known X-linked female-limited disease gene. Subsequent studies have further confirmed the presence of ARR3-related MYP26 (Niu et al., 2024; Széll et al., 2021). A cohort analysis by Wang et al. indicated that among 928 families with eoHM, the ARR3 gene is the most common Mendelian pathogenic gene, accounting for approximately 3.1% (Wang X. et al., 2023). These findings emphasize ARR3’s critical role in eoHM and highlight its potential as a key focus for understanding the genetic basis of high myopia.

Therefore, an in-depth investigation into the role of the ARR3 gene in myopia, particularly its high frequency and unique X-linked female-limited inheritance pattern, is crucial for elucidating the pathogenesis of myopia. This article will review the latest progress in research on the ARR3 gene in myopia, summarize its role in disease mechanisms, and explore future research directions.

### Overview of the arrestin family

**FIGURE 1:**
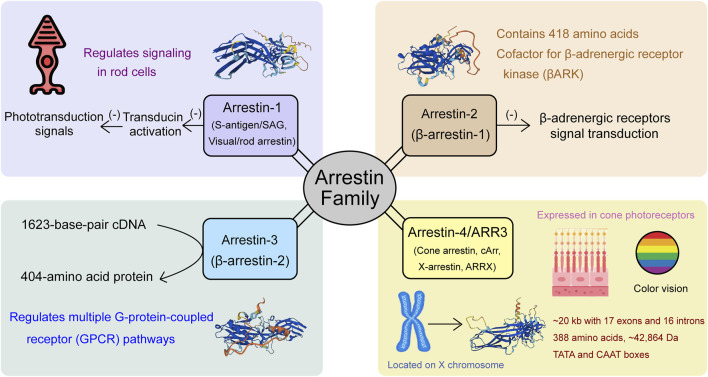
Members of the arrestin gene family: Arrestin-1, Arrestin-2, Arrestin-3 (ARR3), and Arrestin-4, and their functional roles in visual signaling and cellular processes.

Arrestins, originally identified as regulators of signal transduction and receptor desensitization, constitute a highly conserved protein family crucial in regulating G-protein-coupled receptors (GPCRs). In mammals, the arrestin family comprises four distinct members (Figure 1): arrestin-1, β-arrestin-1 (also known as arrestin-2), β-arrestin-2 (also called arrestin-3), and arrestin-4 (cone arrestin, also known as ARR3). Each member plays a distinct role in cellular signaling. Arrestin-1, initially identified as S-antigen or visual/rod arrestin, interacts with phosphorylated rhodopsin in the retina to inhibit transducin activation, thereby terminating the phototransduction signal (Kühn et al., 1984; Wilden et al., 1986). This discovery paved the way for a deeper understanding of the roles of arrestins in receptor regulation. β-arrestins exist in two forms: β-arrestin-1 (arrestin-2), a 418-amino acid protein homologous to retinal arrestin, acts as a cofactor for β-adrenergic receptor kinase (βARK), inhibiting the signal transduction of β-adrenergic receptors phosphorylated by βARK without affecting rhodopsin signaling (Lohse et al., 1990). β-arrestin-2 (arrestin-3 or hTHY-ARRX) is encoded by a 1623-base-pair cDNA, producing a 404-amino acid protein with a molecular weight of 45,275 Da (Shinohara et al., 1987). ARR3 (arrestin-3/cone arrestin) is specifically expressed in cone photoreceptors, essential for color vision, distinguishing it from other arrestin family members. We would like to use ARR3 (OMIM: 301770) to avoid the confusion in the nomenclature.

### ARR3: a novel member of the arrestin family

ARR3, originally referred to as X-arrestin, cArr, or ARRX, is located on the X chromosome, mapping between the Xcen and Xq22 regions. The protein sequence analysis revealed that ARR3 shares significant similarity with other members of the arrestin family, confirming its classification within this group. ARR3 was first cloned from human retina cDNA using subtractive cDNA cloning strategies (Murakami et al., 1993) and the arrestin-homology cloning approach proposed by Craft (Craft et al., 1994). The nearly full-length cDNA sequence consists of 1,314 base pairs and contains a polyadenylation signal at position 1,269, followed by a poly(A) tail at the 3′ end. The open reading frame initiates at position 52 with an ATG start codon, known for its optimal sequence for translation initiation, and terminates at position 1,216 with a stop codon, ultimately encoding a protein of 388 amino acids with an estimated molecular mass of 42,864 Da (Murakami et al., 1993). Sakuma’s investigation into the gene structure of ARR3 revealed that it spans approximately 20 kb and comprises 17 exons and 16 introns. The exons are relatively small, with exon 16 consisting of only 10 bp, and they are organized into three groups separated by the two largest introns (exons 1–5, exons 6–12, and exons 13–17). Despite differences in overall size, notable structural similarities between the ARR3 and S-arrestin genes, particularly in that 10 exons share identical sizes and both genes display similar exon-intron arrangements, suggesting a possible evolutionary or functional relationship. However, significant differences were observed in their promoter sequences. The 5′ upstream region of the ARR3 gene contains TATA and CAAT boxes, characteristic of genes with tissue-specific expression, whereas the S-arrestin gene lacks these elements. Moreover, the shared promoter elements between ARR3 and cone opsin genes, including the TATA box, PCE-1, and CRX-binding sequences, likely play a crucial role in driving cone-specific expression (Sakuma et al., 1998).

Despite sharing 58% sequence homology with bovine β-arrestin and 49%–50% identity with S-antigen, significant differences are evident in the carboxyl-terminal region between β-arrestin and retinal arrestin, as demonstrated by multiple sequence alignments. By preparing affinity-purified anti-peptide antibodies against human ARR3, Sakuma successfully demonstrated labeling of cone photoreceptors, with the highest immunofluorescence intensity observed in their outer segments. Additionally, single and double-labeling experiments confirmed that ARR3 is expressed in red, green, and blue-sensitive cones, further distinguishing it from β-arrestin, which primarily interacts with β-adrenergic receptors, and S-antigen, which is involved in rod photoreceptors (Sakuma et al., 1996). Furthermore, Maeda attempted to isolate the cDNA clone of ARR3 from a bovine retinal cDNA library using a human ARR3 cDNA probe and prepared affinity-purified anti-peptide antibodies based on the specificity of its C-terminus (Palczewski et al., 1991). The results indicated that ARR3 did not bind to rhodopsin under any conditions, including bleaching and/or phosphorylation, suggesting that ARR3 may exclusively require cone visual pigments for receptor binding (Maeda et al., 2000). Overall, these findings position ARR3 as a novel and distinctive member of the arrestin family, highlighting its unique functional and structural characteristics.

### Functional exploration of ARR3 in mouse models of cone photoreceptor activity

Arrestins inhibit the activity of G-protein coupled receptors (GPCRs) after they are phosphorylated by G-protein receptor kinases (GRKs) (Gurevich and Gurevich, 2006a; Gurevich and Gurevich, 2006b). Early research using Grk1^−/−^ and Arr1^−/−^ mouse models focused on rod photoreceptors, revealing that both GRK1 and ARR1 are essential for the normal inactivation of rod photoresponses (Chen et al., 1999; Xu et al., 1997). This prompted researchers to hypothesize that the newly discovered ARR3, widely expressed in all cone photoreceptors, might similarly require GRK-mediated phosphorylation for its desensitizing function. Zhu used Nrl−/− mice to reveal that mouse cone S- and M-opsins illuminated *in vivo* were indeed phosphorylated and bound to ARR3. They also found that in the absence of Grk1, the only GRK expressed in mouse cone photoreceptors, neither phosphorylation of cone opsins nor binding of ARR3 was detectable in Nrl−/−Grk1^−/−^ mice (Zhu et al., 2003). Considering that normal cone inactivation in mice requires Grk1, as demonstrated by recording S- and M-opsin light responses in single cones of Nrl−/− mice (Nikonov et al., 2005), Nikonov further investigated the role of ARR3 by constructing an Arr3^−/−^ mouse model. In contrast to wild-type (WT) mice, it was surprising to find that mouse cones expressed both ARR3 and ARR1, though ARR3 was present at a much lower level. Specifically, the total ARR1 was estimated to be in an ∼6:1 ratio to cone opsin, about 50-fold higher than ARR3, with ARR3 present at a ratio of ∼1:500 relative to cone opsin. Both ARR3 and ARR1, however, were able to inhibit the activity of light-activated S and M cone opsins (Chan et al., 2007). Additionally, ARR3 in cones, similar to ARR1 in rods, undergoes light-dependent redistribution between the inner and outer segments (Chan et al., 2007), further highlighting the complexity of ARR3’s functional role in cones. Nikonov also constructed Arr1^−/−^ and Arr3^−/−^ double knockout mice. They discovered that normal cone opsin inactivation requires at least one of the two arrestins (ARR1 or ARR3) across a range of flash intensities. They also noted that S- and M-opsins form low-activity photoproducts that decay 70-fold faster than rhodopsin photoproducts in rods, suggesting that cone photoresponses may be faster and shorter-lived compared to those of rods (Zhu et al., 2002). Deming further investigated the retinal phenotypes of Arr3^−/−^ mice compared with age-matched wild-type mice, finding that ARR3 modulates essential functions in high-acuity vision and downstream cellular signaling pathways. These functions could not be compensated for by the co-expression of ARR1, despite its high levels in all mouse cones. Without normal ARR3 expression, cone photoreceptors slowly degenerate with increasing age, making this a valuable model for studying age-related cone dystrophy (Deming et al., 2015a). Furthermore, S-opsin expression was found to be lower in the absence of ARR3 (Deming et al., 2015b). Interestingly, despite the substantial concentration differences between ARR3 and ARR1 in cones, the cone shutoff kinetics were approximately the same in Arr1^−/−^ and Arr3^−/−^ mice, indicating that ARR3 may have a higher affinity for cone opsins than ARR1 and plays an important role in terminating the light-activated signals initiated by cone opsins (Nikonov et al., 2008).

### ARR3 and early-onset high myopia: a genetic landscape of mutations and clinical features

**FIGURE 2:**
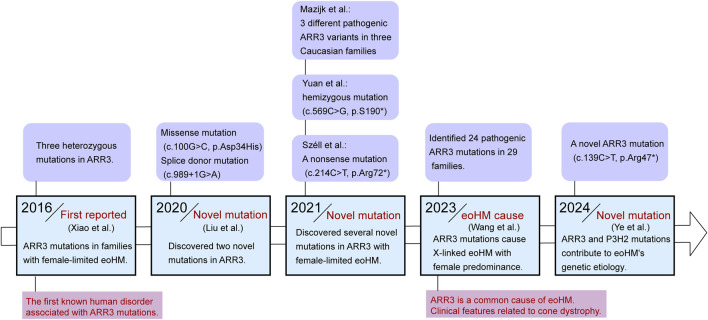
Timeline diagram for the discovery of the relationship between mutations in the ARR3 gene and eoHM.

In 2016, Xiao first identified three different heterozygous mutations in ARR3 across three families with eoHM, with these mutations occurring exclusively in female family members who exhibited the disease. This marked the first instance of a human disorder linked to ARR3 mutations (Xiao et al., 2016). In subsequent years, additional families with ARR3-related early-onset high myopia were identified. In 2020, Liu et al. used whole-exome sequencing (WES) in 67 Tujia Chinese patients with eoHM, discovering both a missense mutation (c.100G>C, p.Asp34His) and a splice donor mutation (c.989+1G>A) in the ARR3 gene (Liu et al., 2020). In 2021, Széll provided the first description of a large Hungarian family displaying female-limited early-onset high myopia, identifying a novel nonsense mutation (c.214C>T, p.Arg72*) in the ARR3 gene (Széll et al., 2021). That same year, Yuan reported a male patient from Southern China with hemizygous ARR3 mutation (ARR3: c.569C>G, p.S190*) associated with high myopia (Yuan et al., 2021). Over the following years, Mazijk in the Netherlands identified three different ARR3 pathogenic variants (c.214C>T, p.Arg72*; c.767 + 1G>A, p.?; c.848delG, p.(Gly283fs)) in three separate Caucasian families (van Mazijk et al., 2022). Ediae reported two individuals with early-onset myopia carrying the c.298C>T/p.Arg100* variant in ARR3 in a multi-generational mixed European/Indigenous Canadian family, a mutation previously identified in an unrelated East Asian family, as well as two additional females from different families (Ediae et al., 2024). Besides, our recent study identified a novel ARR3 mutation (c.139C>T, p.Arg47*) in eoHM families from Shaanxi Province, further expanding the known spectrum of ARR3-related mutations in high myopia (Ye et al., 2024). In addition to these individual family studies, Wang conducted a large-scale study that included 928 families with eoHM, screening for potentially pathogenic variants (PPVs). They identified 24 pathogenic ARR3 mutations in 29 families. This study not only summarized the clinical features of MYP26 patients with ARR3 mutations—highlighting the rarity of typical cone dystrophy symptoms such as photophobia or color blindness—but also noted that macular abnormalities associated with cone dystrophy tend to appear earlier than the myopic maculopathy seen in ARR3-related cases. Moreover, Wang’s study emphasized that ARR3 is one of the most common causes of eoHM (Wang Y. et al., 2023; Figure 2).

Several other studies have since identified ARR3 mutations in additional families with early-onset high myopia, and a summary of these findings can be found in Table 1, which consolidates the mutations and associated clinical characteristics across various reports.

**TABLE 1:** Overview of ARR3 gene mutations identified in early-onset high myopia research.

Year	Author	Title	Country	Gene	Nucleotide	Protein	Type
2016	Xueshan Xiao	X-linked heterozygous mutations in ARR3 cause female-limited early onset high myopia	Chinese (Guangzhou)	ARR3	c.893C>A	p.Ala298Asp	—
					c.298C>T	p.Arg100*	—
					c.239T>C	p.Leu80Pro	—
2020	Fang Liu	Mutation screening of 17 candidate genes in a cohort of 67 probands with early-onset high myopia	Tujia Chinese families	ARR3	c.100G > C	p.Asp34His	Missense
					c.989 + 1G > A	—	Splice
2021	Noémi Széll	Myopia-26, the female-limited form of early-onset high myopia, occurring in a European family	Hungary/Caucasian	ARR3	c.214C>T	p.Arg72*	Nonsense
2021	Dejian Yuan	Identification and Functional Characterization of a Novel Nonsense Variant in ARR3 in a Southern Chinese Family With High Myopia	Southern Chinese	ARR3	c.569C>G	p.S190*	Nonsense
2021	Ralph van Mazijk	Early onset X-linked female limited high myopia in three multigenerational families caused by novel mutations in the ARR3 gene	Netherlands/Caucasian	ARR3	c.214C>T	p.Arg72*	Nonsense
					c.767 + 1G>A	p.?	Splice
					c.848delG	p. (Gly283fs)	Frameshift
2023	Lei Gu	The causal mutation in ARR3 gene for high myopia and progressive color vision defect	Chinese (Zhejiang)	ARR3	c.228T>A	p.Tyr76*	Nonsense
2023	Xuan Xiao	Identification of a Novel Frameshift Variant of ARR3 Related to X-Linked Female-Limited Early-Onset High Myopia and Study on the Effect of X Chromosome Inactivation on the Myopia Severity	Chinese (Wu Han)	ARR3	c.666delC	p. Asn222LysfsTer22	Frameshift
2022	Annechien, E.G., Haarman	Whole exome sequencing of known eye genes reveals genetic causes for high myopia	Netherlands	ARR3	c.484C>T	p. (Arg162Trp)	Missense
					c.767 + 1G>A	p.?	Splice
					c.214C>T	p. (Arg72*)	Nonsense
					c.848delG	p. (Gly283fs)	Frameshift
2023	Min Ye	Mutational investigation of 17 causative genes in a cohort of 113 families with non-syndromic early-onset high myopia in northwestern China	Chinese (Ningxia)	ARR3	c.767 + 1G > A	—	Splicing
2023	Grace Uwaila Ediae	Pathogenic variant in the X-linked ARR3 gene associated with variable early-onset myopia	Canada (Ontario)	ARR3	c.298C>T	p.Arg100*	Nonsense
2024	Jianing Niu	Novel Splicing Variants in the ARR3 Gene Cause the Female-Limited Early-Onset High Myopia	Chinese (Shanghai)	ARR3	c.39 + 1G>A	p.Leu14Metfs*16	Splicing
					c.100 + 4A>G	p.Asp34Glyfs*16	Splicing
2024	Lu Ye	Trio-based whole-exome sequencing reveals mutations in early- onset high myopia	Chinese (Shaanxi)	ARR3	c.139C>T	p.Arg47*	Missense
2022	Yingwei Wang	Genetic and clinical landscape of ARR3- associated MYP26: the most common cause of Mendelian early-onset high myopia with a unique inheritance	Chinese (Guangzhou)	ARR3	c.3G>A	p.M1I?	
					c.9-1G>A	SA	
					c.103G>A	p.G35S	
					c.139C>T	p.R47*	
					c.146T>G	p.L49W	
					c.149T>C	p.F50S	
					c.232C>T	p.Q78*	
					c.239T>C	p.L80P	
					c.298C>T	p.R100*	
					c.345G>C	p.Q115H	
					c.346–2A>T	SA	
					c.361C>A	p.P121T	
					c.386_389del	4	
					c.499A>T	p.K167*	
					c.520G>T	p.E174*	
					c.520delG	0	
					c.707C>G	p.T236R	
					c.757delC	p.Q253Rfs*7	
					c.844_845insT	p.R282Lfs*10	
					c.893C>A	p.A298D	
					c.928G>T	p.E310*	
					c.929_930del	6	
					c.963_964del	5	
					c.1014–2A>G	SA	

### Female-limited inheritance of ARR3 variants in early-onset high myopia

In 2016, Xiao identified ARR3 gene variants associated with female-limited early-onset high myopia (Myopia 26), demonstrating a sex-limited inheritance pattern where heterozygous females are primarily affected, while hemizygous males generally remain unaffected or exhibit only mild symptoms (Xiao et al., 2016). This distinctive sex-specific manifestation parallels other X-linked conditions, such as craniofrontonasal syndrome and epilepsy caused by mutations in EFNB1 (Twigg et al., 2004) and PCDH19 (Jamal et al., 2010), where heterozygous females are also more severely impacted than their hemizygous male counterparts.

The underlying mechanism for this female-predominant phenotype likely involves the complex regulation of X-linked genes and epigenetic factors, notably X-chromosome inactivation (XCI). XCI functions to balance gene dosage between males (XY) and females (XX) by randomly inactivating one X chromosome in females, resulting in mosaic expression of the gene in question (Patrat et al., 2020). Consequently, heterozygous females with ARR3 variants may express a mixture of wild-type and mutant alleles, contributing to the variability in phenotypic expression. In the study by Xiao, methylation analysis of XCI patterns revealed that the proband had an XCI rate of 24.37% on the X chromosome carrying the ARR3 mutation, indicating predominant expression of the mutant allele and correlating with a severe phenotype. Conversely, the proband’s mother, with an XCI ratio of 81.30%, displayed only mild symptoms, while her sister, with an inactivation rate of 56.12%, exhibited high myopia without early onset, thus reflecting an intermediate phenotype severity. This pattern was consistently observed in two additional family members who also carried the ARR3 mutation, each displaying mild phenotypes without the hallmark early-onset of high myopia (Xiao et al., 2023).

These findings suggest a potential correlation between phenotype severity and the proportion of mutant-expressing cells, which may explain the phenotypic heterogeneity observed among family members carrying the same ARR3 variant.

### Potential pathogenic mechanisms of ARR3 mutations in early-onset high myopia

It remains unclear how pathogenic variants in ARR3 lead to myopia. Széll e conducted an in-depth exploration of potential mechanisms in their study (Széll et al., 2021). They posited that defects in ARR3 could result in limited arrestin function in L/M cones. Gu, using 10 years of clinical follow-up data, identified gradually worsening cone dysfunction and color vision as key features among individuals affected by ARR3 mutations, further supporting the hypothesis of cone cell involvement (Barboni et al., 2024). Consequently, abnormalities in ARR3 may lead to increased opsin activity in L/M cones, heightening sensitivity to red/green visual stimuli. This heightened sensitivity can exacerbate chromatic aberrations, causing short-wavelength light (such as blue light) to focus in front of the retina, while long-wavelength light focuses behind it (Nir and Ransom, 1992). This phenomenon, along with excessive accommodation and axial elongation, contributes to increased luminance contrast, which are both critical features in the pathogenesis of myopia (Rucker and Kruger, 2006). Furthermore, this transitional over-accommodation may result in hyperopic defocus, where images form behind the retina, inducing ocular elongation and worsening myopia progression (Erdinest et al., 2023).

Additionally, blue light has been recognized as having significant regulatory effects on ocular development. It is believed to inhibit abnormal eye growth and prevent the onset of myopia (Wang X. et al., 2023). Mutations in ARR3 may impair the function of arrestin in cone cells, particularly in L and M cones, which could diminish their responses to blue light. This impairment could reduce blue light transmission within the retina, weakening its protective effect against myopia and making the eye more susceptible to elongation during periods of over-accommodation and prolonged defocus (Rucker et al., 2015).

The ganglion cell hypothesis posits that retinal ganglion cell (RGC) dysfunction, particularly in intrinsically photosensitive retinal ganglion cells (ipRGCs), plays a significant role in the development of refractive errors (Kinder et al., 2022). Specifically, the melanopsin-driven circadian clock within the retina, maintained by intrinsically photosensitive retinal ganglion cells (ipRGCs), which are primarily responsible for non-image-forming visual functions like circadian rhythms and pupil responses, plays a crucial role in regulating emmetropization (Chakraborty et al., 2018; Ecker et al., 2010). Recent study by Barboni, have provided empirical evidence for this dysfunction in myopia associated with ARR3 (Barboni et al., 2024). In their study, they observed that patients with the female-limited form of early-onset high myopia associated with ARR3 exhibited a significantly reduced postillumination pupillary response (PIPR) to blue light compared to control groups. This response, which is primarily mediated by melanopsin in ipRGCs, reflects the cell’s ability to maintain pupil constriction after light exposure, particularly to short-wavelength blue light. The diminished PIPR in these eoHM patients not only highlights a clear dysfunction in the melanopsin-mediated ipRGC system, but also indicates that this system is not functioning optimally in individuals with ARR3 mutations. This malfunction may contribute to pathological eye elongation in MYP-26, as ipRGCs are critical in regulating eye growth through light-dependent mechanisms. Furthermore, ipRGC dysfunction could also disrupt dopaminergic signaling, as these cells are connected to dopaminergic amacrine cells in the retina. This disruption may reduce dopaminergic signaling due to impaired synaptic connections between ipRGCs and dopaminergic amacrine cells, thereby diminishing dopamine’s protective role against myopia (Dkhissi-Benyahya et al., 2013; Wang et al., 2018).

**FIGURE 3:**
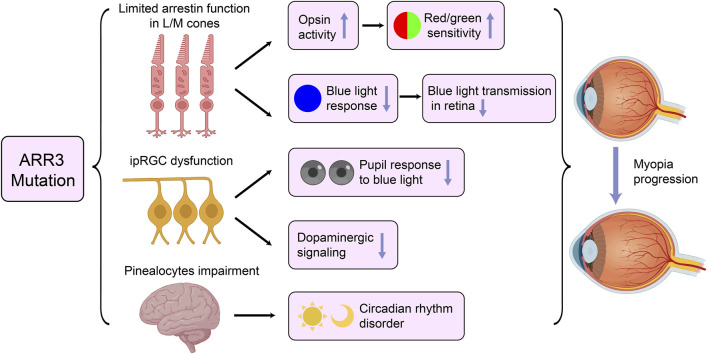
Potential Pathogenic Mechanisms of ARR3 Mutations in Early-Onset High Myopia. ipRGC, intrinsically photosensitive retinal ganglion cells.

Moreover, ipRGCs regulate circadian rhythms through connections with the suprachiasmatic nucleus (SCN) and pineal gland, which can affect melatonin release (Chakraborty et al., 2018). Disruptions in circadian rhythms have been linked to eye elongation and myopia progression (Stone et al., 2019). ARR3 mutations may lead to functional impairments in pinealocytes, which share key arrestin family proteins with retinal photoreceptor cells (Sunayashiki-Kusuzaki et al., 1997). Such impairments could alter melatonin production, leading to disturbances in circadian rhythms that may, in turn, contribute to the progression of myopia. Given the multifaceted role of ARR3 in both visual signaling and circadian regulation, further investigation is needed to fully understand its impact on myopia progression. This requires exploring the complex interactions between retinal and systemic pathways. For a more detailed illustration of the relationship between ARR3 mutations and myopia, please refer to Figure 3.

## Discussion

As myopia has become a global public health concern, exploring its pathogenic factors is increasingly critical (Morgan et al., 2021). Since ARR3 was first identified as a causative gene for early-onset high myopia in 2016 (Xiao et al., 2016), growing research has revealed its strong association with myopia, positioning it as one of the most prevalent myopia-related pathogenic genes (Wang Y. et al., 2023). Compared to other non-syndromic myopia-associated genes, ARR3 exhibits several distinct characteristics. Firstly, the gene is located on the X chromosome, which underlies its X-linked, female-limited inheritance pattern (Murakami et al., 1993). Secondly, ARR3 contains multiple conserved functional domains, and mutations within these domains frequently result in functional abnormalities, thereby enhancing its pathogenic potential (Sakuma et al., 1998). Furthermore, ARR3 serves as a critical regulator of phototransduction, with its expression predominantly confined to cone photoreceptors, where it plays an indispensable role in the signal transduction process (Maeda et al., 2000; Sakuma et al., 1996). Specifically, the ARR3 gene product binds to light-activated, phosphorylated receptors, terminating phototransduction in photoreceptors (Nikonov et al., 2008). Therefore, ARR3’s pathogenic mechanism likely influences refractive status indirectly via cone cell dysfunction, rather than through direct effects on ocular structural development, scleral growth, or corneal curvature. Given the unique inheritance pattern of ARR3 and its critical role in phototransduction, this gene holds significant value for further investigation in understanding the pathogenesis of myopia, particularly early-onset high myopia.

Extensive research on ARR3 mutations in eoHM has primarily been conducted within various Chinese populations, encompassing both regional cohorts, such as those from Wuhan (Xiao et al., 2023), Ningxia (Ye et al., 2023), and Shanghai (Niu et al., 2024), as well as studies focused on specific ethnic groups, including Tujia Chinese families (Liu et al., 2020). These studies have identified a broad spectrum of mutations across various regions and ethnicities in China, highlighting the genetic heterogeneity of eoHM and underscoring the pivotal role of ARR3 in the pathogenesis of myopia. However, the genetic landscape of ARR3-related eoHM extends beyond Chinese populations. Investigations from European cohorts, including those from the Netherlands (van Mazijk et al., 2022) and Hungary (Széll et al., 2021) have also uncovered a diverse array of ARR3 mutations, albeit at a lower frequency. Notwithstanding these regional and ethnic disparities, a significant degree of overlap exists in the specific mutations identified, pointing to a common pathogenic mechanism across different populations. For instance, Ediae et al. (2024) reported the c.298C>T (p.Arg100*) variant in ARR3, found in a multi-generational European/Indigenous Canadian family (Ediae et al., 2024), which mirrors the mutations observed in Chinese cohorts, including those from the Zhongshan population (Wang X. et al., 2023). This observation suggests that certain ARR3 mutations may be broadly applicable across ethnicities, emphasizing the potential for shared genetic factors underlying eoHM.

### Further insights into XCI and ARR3-associated inheritance

The X chromosome carries 3,637 known protein-coding genes (Information 2024). Variations in some of these non-lethal genes contribute to at least 577 X-linked disorders that disproportionately affect males more severely (OMIM 2024), such as mutations in FGD1 (Xp11.254,445,453), leading to Aarskog–Scott syndrome allelic with X-linked intellectual disability 16 (XLMR 16). In affected males, this syndrome manifests with facial and genital abnormalities (such as hypertelorism and shawl scrotum), attention deficit hyperactivity disorder (ADHD), and intellectual disability, while females exhibit only mild traits, such as a widow’s peak hairline or short stature (Li et al., 2024; Zhu et al., 2022). Most of these X-linked genes, however, do not influence sexual development directly but rather play roles in non-reproductive tissues, including the retina. In the current study, ARR3 mutations associated with eoHM exhibited heightened susceptibility and more severe symptoms in females. Considering the complexity of the X chromosome, this inheritance model may also involve epigenetic regulation, with X-chromosome inactivation potentially offering part of the explanation. In female mammals, somatic cells can recognize the number of X chromosomes present and transcriptionally silence all but one in each set of autosomes, equalizing X-linked gene dosage between females (XX) and males (XY). Once XCI is established, it is stably maintained through epigenetic remodeling and transmitted through mitosis, ensuring that each cell contains one active X chromosome (Xa) and one inactive X chromosome (Xi) (Lyon, 1962). As a result, females with X-linked disorders often experience milder symptoms since the mutation is expressed in only half of their cells. In the context of myopia, however, ARR3’s role in phototransduction in cone cells could increase photosensitivity and enhance contrast differences, potentially creating retinal image disparities that promote myopia development.

It is also noteworthy that in normal female populations, the XCI pattern shows substantial variability. When this ratio deviates significantly, it is referred to as X-chromosome inactivation skewing (Vacca et al., 2016). This unbalanced XCI pattern can occur through selective proliferation of certain cells after an initial, random XCI establishment or may be influenced by genetic factors, suggesting that the initial XCI choice may not be entirely random (Clerc and Avner, 2006). This could potentially explain why other female carriers with ARR3 mutations in the same eoHM family exhibit atypical eoHM presentations, where the degree of inactivation of the mutant allele is correlated with the myopia phenotype (Martínez et al., 2005; Xiao et al., 2023).

Meanwhile, male family members carrying the pathogenic ARR3 mutation present with astigmatism and mild myopia but do not exhibit eoHM, show a later age of onset (van Mazijk et al., 2022), or display no myopia phenotype at all (Xiao et al., 2023). In the study by Yuan, it was observed that the proband’s father, affected by high myopia (HM), possessed this pathogenic ARR3 mutation. Interestingly, an ARR3 nonsense mutation was identified in affected male hemizygotes, while unaffected male hemizygotes in other families carried missense mutations. This suggests that the mechanism by which null variants cause functional loss may be more detrimental to the ARR3 gene (Yuan et al., 2021). Moreover, mouse studies have shown that ARR1 and ARR3 can compensate for each other in cone cells, with the knockout of either gene alone only mildly affecting recovery time following intense light exposure. However, knocking out both genes leads to a substantial delay (Nikonov et al., 2008). This functional redundancy may explain the milder phenotypes in males with certain ARR3 variants, as ARR1 might compensate for partial ARR3 loss. Thus, although these specific cases warrant further investigation, they do not definitively negate the X-linked female-limited inheritance pattern for ARR3-associated high myopia.

### Elaborating on the mechanisms of ARR3 in myopia pathogenesis

The accommodative response is highly sensitive to the luminance and chromatic components of a stimulus (Rucker and Kruger, 2004a; Rucker and Kruger, 2004b). Both luminance and chromatic components elicit responses with distinct temporal characteristics, and the focal properties of these responses depend on chromatic changes in the retinal image due to longitudinal chromatic aberration (LCA). LCA signals arise from the dispersion of white light by optical media, providing defocus direction information when sampled at a single focal plane (Aggarwala et al., 1995a; Aggarwala et al., 1995b). Specifically, chromatic short-wavelength cone contrast biases the response towards near focus, while greater long-wavelength contrast relative to middle-wavelength contrast biases the accommodation response toward distance. Additionally, the response time of short-wavelength chromatic cones to a step change in focus is approximately three times slower compared to when luminance contrast is present (Rucker and Kruger, 2004b). Thus, individual sensitivity to the luminance and chromatic components of a stimulus affects both the timing and focal characteristics of the accommodative response, potentially predisposing individuals to myopia development. Rucker’s studies further confirmed this by measuring accommodation under various cone contrast ratios; she found that both the mean accommodation level and the gain of the accommodative response to sinusoidal stimulus movements depend on the relative sensitivity of L and M cones. When luminance contrast is maximized through accommodation, long wavelengths are focused behind the photoreceptors. In individuals whose luminance response is dominated by L-cones, both increased accommodation and ocular elongation could enhance luminance contrast, resulting in myopia (Rucker and Kruger, 2006). Mutations in ARR3, which disrupt LM cone functionality, may enhance their sensitivity, fostering greater accommodative responses and providing a foundation for myopia onset (Barboni et al., 2024).

Furthermore, previous studies indicate that S-cones contribute to accommodation responses, suggesting that the myopically defocused blue light provides a stimulus for reduced eye growth (Kröger and Wagner, 1996; Rucker and Kruger, 2001; Seidemann and Schaeffel, 2002). Studies on chick models, which share similar spectral sensitivity and temporal responses with humans, have examined the effects of blue light on eye growth. The findings indicate that light sources rich in blue light can protect against myopic eye growth when the eye is exposed to slow changes in luminance. At lower temporal frequencies, the visual system has sufficient time to detect myopically defocused blue light, responding with slower growth in the presence of blue light (Rucker et al., 2015). In patients with ARR3 mutations, although ARR1 is the primary arrestin family member expressed in S-cones, ARR3 dysfunction may lead to excessive activation of L and M cones. This overactivation could disrupt the balance of visual signals in cone cells, thereby diminishing the response to blue light defocus and weakening the protective effect of blue light in myopia control (Rucker et al., 2015).

The ganglion cell hypothesis attributes the development of refractive errors to the dysfunction of ipRGCs (Aranda and Schmidt, 2021). These cells serve a dual role in visual processing: they transmit visual information from photoreceptors to higher visual centers and directly detect light through their photosensitive protein, melanopsin. This capability allows ipRGCs to transduce signals from both rod and cone photoreceptors, similar to classical RGCs (Do, 2019; Graham and Wong, 1995). Furthermore, classical RGCs and ipRGCs are interconnected horizontally via amacrine cells, facilitating interactions between these cell types (Müller et al., 2010; Vuong et al., 2015). In addition to their recognized role in non-image-forming visual functions—such as circadian rhythm regulations (Hartmann et al., 2021) and pupil responses (Mostafa et al., 2021)—they have also recently been identified as contributors to conscious, detail-oriented visual processing (Ecker et al., 2010; Matynia, 2013). All these functions are related to the occurrence and progression of myopia. Notably, while RGC dysfunction is observed on pattern electroretinography (PERG) in patients (Széll et al., 2021), further research is needed to elucidate how this impairment may relate to both ARR3 mutations and specific hereditary features.

In conclusion, the investigation of ARR3 and its multifaceted role in myopia highlights the need for continued research into the genetic and molecular underpinnings of this condition. Understanding these mechanisms may pave the way for targeted interventions and improved management strategies for individuals affected by myopia, particularly early-onset high myopia.
